# Comparative genomics of *Glandirana rugosa* using unsupervised AI reveals a high CG frequency

**DOI:** 10.26508/lsa.202000905

**Published:** 2021-03-12

**Authors:** Yukako Katsura, Toshimichi Ikemura, Rei Kajitani, Atsushi Toyoda, Takehiko Itoh, Mitsuaki Ogata, Ikuo Miura, Kennosuke Wada, Yoshiko Wada, Yoko Satta

**Affiliations:** 1 Primate Research Institute, Kyoto University, Inuyama-shi, Japan; 2 Amphibian Research Center, Hiroshima University, Hiroshima-shi, Japan; 3 Department of Evolutionary Studies of Biosystems, School of Advanced Sciences, The Graduate University For Advanced Studies (SOKENDAI), Shonankokuraimura, Hayama-machi, Japan; 4 Department of Bioscience, Nagahama Institute of Bio-Science and Technology, Nagahama-shi, Japan; 5 Department of Life Science and Technology, School of Life Science and Technology, Tokyo Institute of Technology, Tokyo-to, Japan; 6 Department of Genomics and Evolutionary Biology, National Institute of Genetics, Mishima-shi, Japan; 7 Preservation and Research Center, Yokohama, Japan

## Abstract

The Japanese wrinkled frog (*Glandirana rugosa*) is unique in having both XX-XY and ZZ-ZW types of sex chromosomes within the species. The genome sequencing and comparative genomics with other frogs should be important to understand mechanisms of turnover of sex chromosomes within one species or during a short period. In this study, we analyzed the newly sequenced genome of *G. rugosa* using a batch-learning self-organizing map which is unsupervised artificial intelligence for oligonucleotide compositions. To clarify genome characteristics of *G. rugosa*, we compared its short oligonucleotide compositions in all 1-Mb genomic fragments with those of other six frog species (*Pyxicephalus adspersus*, *Rhinella marina*, *Spea multiplicata*, *Leptobrachium leishanense*, *Xenopus laevis*, and *Xenopus tropicalis*). In *G. rugosa*, we found an Mb-level large size of repeat sequences having a high identity with the W chromosome of the African bullfrog (*P. adspersus*). Our study concluded that *G. rugosa* has unique genome characteristics with a high CG frequency, and its genome is assumed to heterochromatinize a large size of genome via methylataion of CG.

## Abstract (precis)

Genome sequencing of a unique frog (*Glandirana rugosa*) having XY/ZW systems within the species and comparative genomics with other six frogs were performed using a batch-learning self-organizing map, which is unsupervised AI for oligonucleotide compositions, to clarify its genome characteristics.

## Introduction

The sex determination system (XX-XY or ZZ-ZW) is known to change often during evolution of organisms, and the systems in amphibians and fishes have changed at a higher rate than that in mammals and birds (Bachtrog et al, 2014). In particular, the Japanese wrinkled frog (*Glandirana rugosa*) is unique in having both XX-XY and ZZ-ZW systems within the species (Miura, 2007, 2017), indicating that alteration of the systems is still ongoing (Ogata et al, 2018); for details of the sex determination system of *G. rugosa*, see the Discussion section. To clarify its genome characteristics, we have decoded the genome sequence of *G. rugosa* which is a diploid species, but whose assembled genome size (7.08 Gb) is larger than that of the tetraploid *Xenopus laevis* (2.7 Gb; Session et al, 2016; Li et al, 2019) and ∼4.5 times larger than that of the evolutionarily related diploid *Pyxicephalus adspersus* (1.56 Gb; Denton et al, 2018 *Preprint*). The large *G. rugosa* genome is due not to whole genome duplication, but probably because of explosive proliferation of transposons or partial genome duplications. In a large genome, it is conceivable that expression in a wide area of the genome is suppressed, and comparisons with the small or different size of genomes should yield knowledge about the molecular mechanisms of the suppression in a wide genomic range. A research project aimed at complete genome sequencing of *G. rugosa*, including an advanced assembly, gene annotations, and chromosomal attributions, is still underway, but the present study focused on the analysis of species-specific genomic characteristics and searched for possible structures involved in sex chromosomes.

To understand the *G. rugosa* genome characteristics by comparing its large genome with other frog ones, the present study introduces a new strategy that uses unsupervised artificial intelligence (AI) because AI (machine learning) has become an essential technology for efficient data mining from large and complex data. Notably, unsupervised AI can discover new knowledge without particular models or prior knowledge and is highly desirable for unveiling characteristics hidden in the data; this is a data-driven research based on findings by the unsupervised AI. Specifically, comparative genomics was performed by the batch-learning self-organizing map (BLSOM) using short oligonucleotides (Abe et al, 2003). The short oligonucleotide composition is unique characteristics to each species and is often described as a “genome signature” meaning a characteristic frequency of oligonucleotides (Karlin, 1998); importantly, even if the genome is fragmented (e.g., to 100 kb), most of the fragments have a similar oligonucleotide composition. The genome signature of many species has been visualized easily by the BLSOM (Abe et al, 2005, 2006; Nakao et al, 2013). Using the oligonucleotide BLSOM, we previously analyzed the human genome and found a large Mb-level structure consisting of repetitive sequences rich in CG (the Mb-level CpG island) in centromeric and pericentromeric heterochromatin, as well as in subtelomeric regions (Wada et al, 2015, 2020). In this study, we also found Mb-level large CpG islands on frog genomes by comparative genomics using seven species (*G. rugosa*, *P. adspersus*, *Rhinella marina*, *Spea multiplicata*, *Leptobrachium leishanense*, *X. laevis*, and *Xenopus tropicalis*) and showed that *G. rugosa* had genome characteristics with a high CG frequency.

## Results

### BLSOM analysis

**Figure 1.**
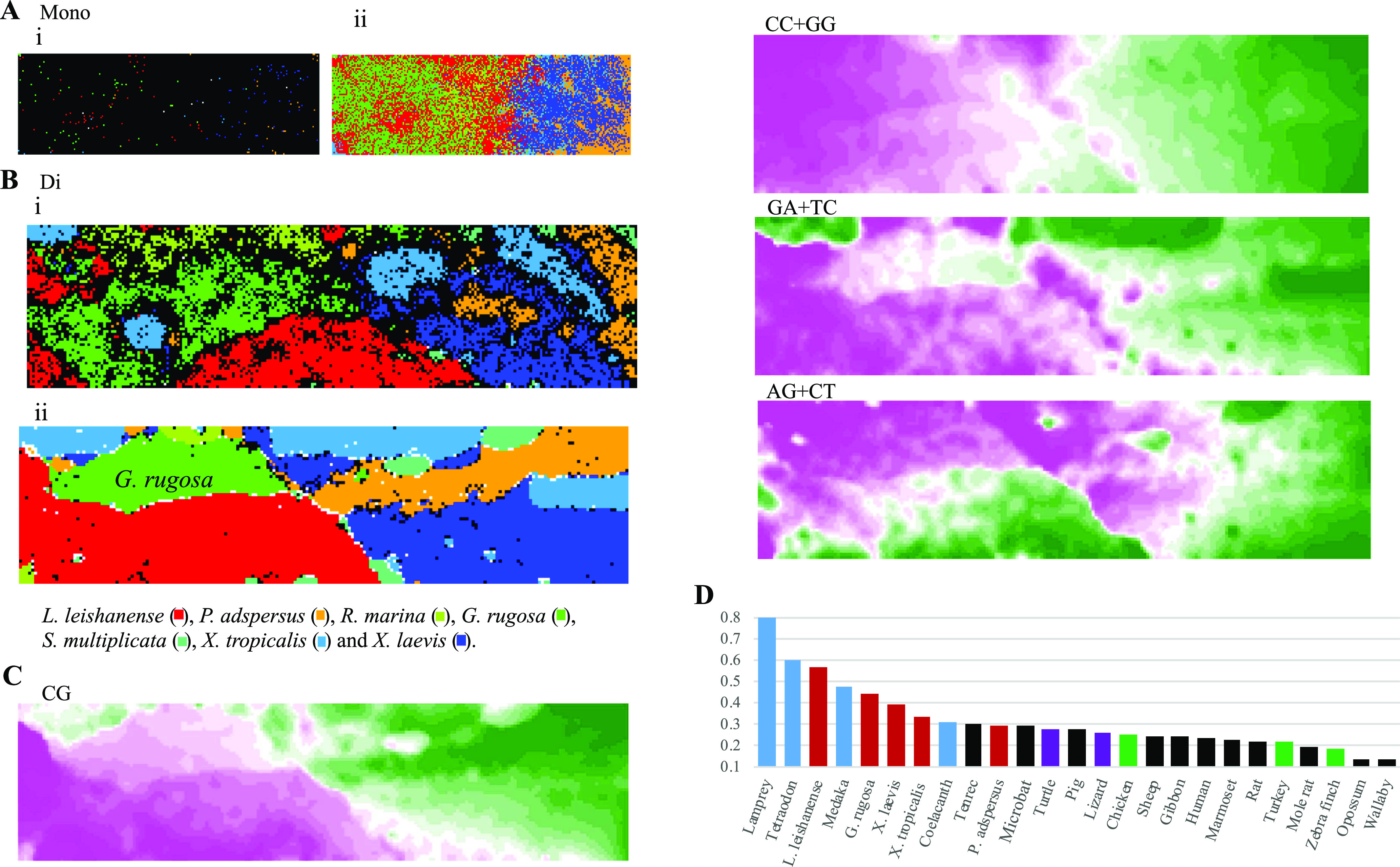
Batch-learning self-organizing maps (BLSOMs) and normalized CG levels. **(Ai, Aii)** Mononucleotide (Mono) BLSOMs for 100-kb frog sequences. Nodes containing sequences from more than one species are indicated in black, and those containing sequences only from one species are indicated in color to distinguish species; nodes that do not contain sequnces after machine learning were left as blank (white) (Ai). When sequences of a single species occupy more than 50% at a node, the color indicating that species is given (Aii). **(Bi, Bii)** Dinucleotide (Di) BLSOMs for 100-kb sequences and 1-Mb sequences sliding with a 100-kb step, respectively. Nodes are marked as described in Ai. **(C)** The contribution level of each dinucleotide to each node is visualized by a color: pink (high), white (moderate), and green (low); results of all dinucleotides are presented in Fig S2. **(D)** Normalized CG levels of 25 vertebrates are arranged in the descending order: fishes (blue), frogs (reddish brown), reptiles (violet) and mammals (black).

**Figure S1.**
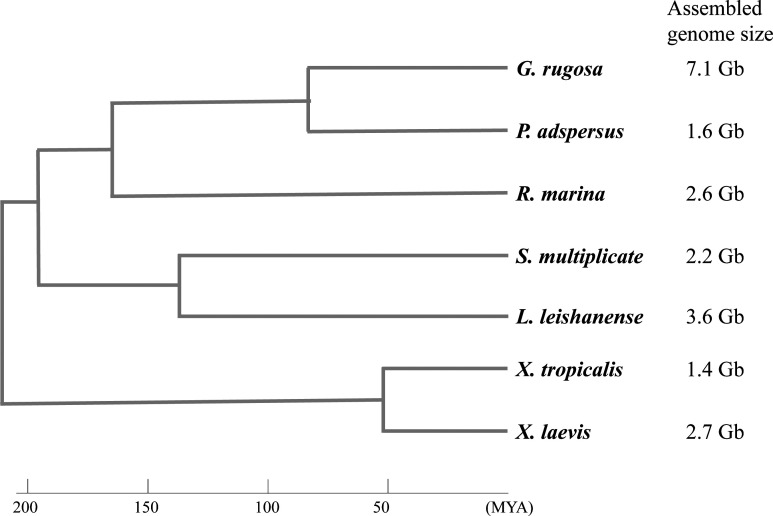
The phylogeny and assembled genome sizes of seven frogs used in this study. The phylogenetic relationship of the frogs is derived from the tree on AmphibiaWeb (https://amphibiaweb.org), and the divergence time scale shown at the bottom is based on times estimated using TIMETREE (http://www.timetree.org). The genome size of each frog was estimated in this study (*Glandirana rugosa*) and in previous studies of other groups: Denton et al (2018) *Preprint* (*Pyxicephalus adspersu*s), Seidl et al (2019) (*Spea multiplicata*), Li et al (2019) (*Leptobrachium leishanense*), Hellsten et al (2010) (*Xenopus tropicalis*), and Session et al (2016) (*Xenopus laevis*). The assembled genome size of *Rhinella marina* was estimated to be in the range of 1.98–2.38 Gb (Edwards et al, 2018), and the average of the range is shown here.

We analyzed the short oligonucleotide composition in genomes of seven frogs, including *G. rugosa* using the BLSOM (Fig 1); the current status of genome sequencing of *G. rugosa* is explained in the Materials and Methods section. Because the oligonucleotide composition inevitably depends on mononucleotide compositions, we first constructed a BLSOM with the mononucleotide composition of all 100-kb sequences derived from the seven genomes (Figs 1A and S1). The total number of nodes (grid points) was set to 1/10 of the total number of sequences (144,623); each node thus has an average of 10 sequences. In Fig 1Ai, grid points containing sequences of a single species are colored to indicate each species, and grid points containing sequences of multiple species are displayed in black. Most points are black, showing that the sequences are not separated accurately by species. Next, when sequences of a single species occupy more than 50% at a grid point, the color indicating that species is given (Fig 1Aii), and this shows that the mononucleotide composition differs among species even when fragmented to 100 kb; *G. rugosa* and *L. leishanense* with high genome G+C% among the frog genomes (44.5% and 43.4%, respectively) are located on the left side in the map (Fig 1Aii), but their sequences are intermingled there; *X. laevis* and *P. adspersus* with low genome G+C% (38.5% and 37.9%, respectively) are on the right side.

Each genome consists of double strands, but only one-strand sequence is registered by The International Nucleotide Sequence Database Collaboration; in the case of a scaffold sequence, there is arbitrariness as to which strand is registered. Furthermore, when general features of genomic sequences of one species (e.g., genome signature) are considered, there is little meaning in distinguishing complementary sequences. Therefore, a pair of complementary oligonucleotides are summed as a group in the present study; complementary oligonucleotides such as AA and TT are not distinguished, and their occurrences are summed (Abe et al, 2005).

Fig 1Bi shows a BLSOM for the composition of the degenerate sets of dinucleotides under the condition that an average of 10 sequences belongs to each node. Even though species information is not given during the machine learning, a large portion of 100-kb sequences have clustered (self-organized) in the territories of each species and thus colored. Next, Fig 1Bii shows a BLSOM with the dinucleotide composition in all 1-Mb sequences sliding with 100-kb width. Whereas the average number of sequences belonging to one node is almost the same as that in Fig 1Bi (10 sequences per node), separation into the species-specific colored territories becomes far clearer than that of the 100-kb BLSOM, making it easier to detect species-specific characteristics, such as genome signature. The subsequent BLSOM analyses, therefore, focus on the 1-Mb sequences sliding with 100-kb width. On the map of Fig 1Bii, *L. leishanense* (3.56 Gb; Li et al, 2019) and *G. rugosa*, which have particularly large assembled genomes, form their own large territories (red and green, respectively), but *X. tropicalis* (1.44 Gb; Hellsten et al, 2010) and *P. adspersus* (1.56 Gb; Denton et al, 2018 *Preprint*), which have small ones, form multiple territories rather than one large territory. In this study we use the assembled genome or total genome sequence length to compare their genome sizes because a genome size based on nuclear DNA content is not investigated or published yet in some frogs including *G. rugosa*. In the case of frogs (*R. marina* and *S. multiplicata*) whose genome is only partially assembled, tiny territories are scattered often within a large territory of other species (Figs 1Bii and S1).

### Oligonucleotides prominently contributing to species-dependent separations

**Figure S2.**
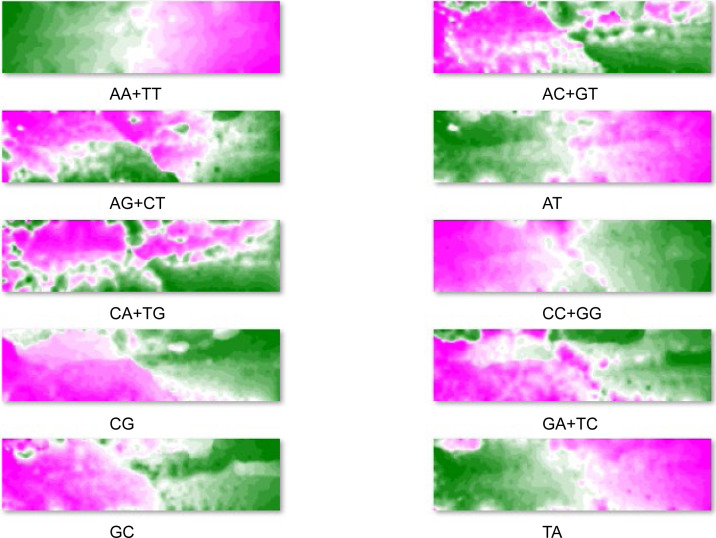
The contribution level of each dinucleotide to each node is visualized on the dinucleotide batch-learning self-organizing map by a color: pink (high), white (moderate), and green (low) as described in Fig 1C.

The BLSOM is an explainable AI and can provide reasons for why a species-dependent separation (self-organization) has occurred. Fig 1C shows four examples of dinucleotides for the BLSOM listed in Fig 1Bii; by focusing on vectorial data (i.e., dinucleotide frequencies) representing each grid point, the grid points are sorted in the descending order of their frequency for each dinucleotide. The descending order of grid points is then represented as a heat map for each dinucleotide; the higher rank points are displayed in pink, the middle ranks in white, and the lower ranks in green (Kanaya et al, 2001); results for all dinucleotides are listed in Fig S2. When searching the heat map pattern that reflects the species-dependent separation, CG shows a good match with the separation (Fig 1C). Specifically, CG is characteristically higher (pink) in the territory of *L. leishanense* and *G. rugosa* than in others (green) (Fig 1C). The frequency of other dinucleotides does not show a clear match with species borders of territories; for example, CC+GG is generally higher on the left side, but does not agree well with the species territories (Fig 1C). Notably, the separation of the dinucleotide BLSOM does not simply reflect an additive effect of the mononucleotide composition; for example, the frequency of GA+TC and AG+CT, which have the same mononucleotide composition, differs among species (Fig 1C).

### Comparison of CG occurrence among species

The basic strategy of this data-driven investigation is to leave knowledge discovery to the unsupervised AI and, based on the obtained knowledge, to conduct more detailed analyses. It is of interest that CG occurs more frequently in *G. rugosa* and *L. leishanense* than in other species (Fig 1C). Methylation at C in CG is a typical epigenetic modification, and the methylated C induces histone deacetylation, subsequent chromatin condensation, and heterochromatinization (Klose et al, 2005; Bogdanović & Veenstra, 2009). Hence, we conduct the detailed analyses of this biologically important dinucleotide in the frog genomes.

**Figure S3.**
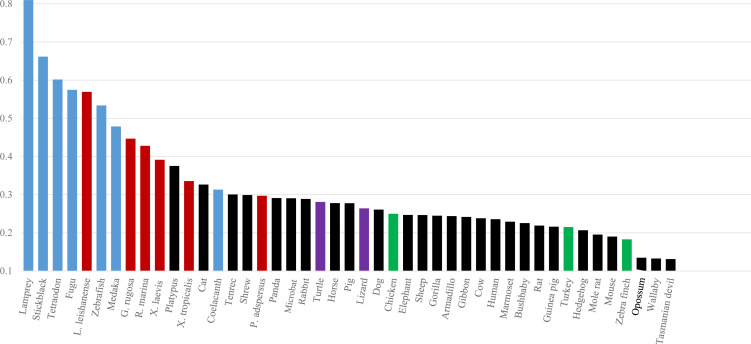
Normalized CG levels of 45 vertebrates are arranged in the descending order: fishes (blue), frogs (reddish brown), reptiles (violet), and mammals (black).

Because methyl-CG tends to mutate to TG/CA, CG is known to occur at a low frequency in vertebrate genomes: that is, CG suppression (Law & Jacobsen, 2010). One index of the CG suppression, which excludes influences of mononucleotide compositions, is the odds ratio of CG which is obtained by dividing the observed occurrence (Obs) of CG by its expected value (Exp) calculated from the mononucleotide composition. Colored bars in Fig 1D show the Obs/Exp values for genomes of 25 vertebrates covering a wide phylogenetic range (those for 45 vertebrates are presented in Fig S3); frog genomes with total reported contig sequences (>1 Mb) >1 Gb are included. The CG suppression is evident in mammals (black), and their Obs/Exp values are distributed in a narrow range (∼0.2–0.3) except for the opossum and wallaby values (<0.2) (Fig 1D). In contrast, the Obs/Exp values for fishes (blue) vary considerably (0.7–0.3); for example, coelacanth is evolutionarily close to tetrapods, and the value for coelacanth is at the same level as that for tetrapods (Iwasaki et al, 2014). The Obs/Exp values for frogs (reddish brown) also vary considerably; the highest is 0.57 for *L. leishanense*, and *G. rugosa* is the second highest at 0.45, but *P. adspersus* is 0.30 which corresponds to the reptile (violet) and mammal levels.

### CG distribution on each chromosome

**Figure 2.**
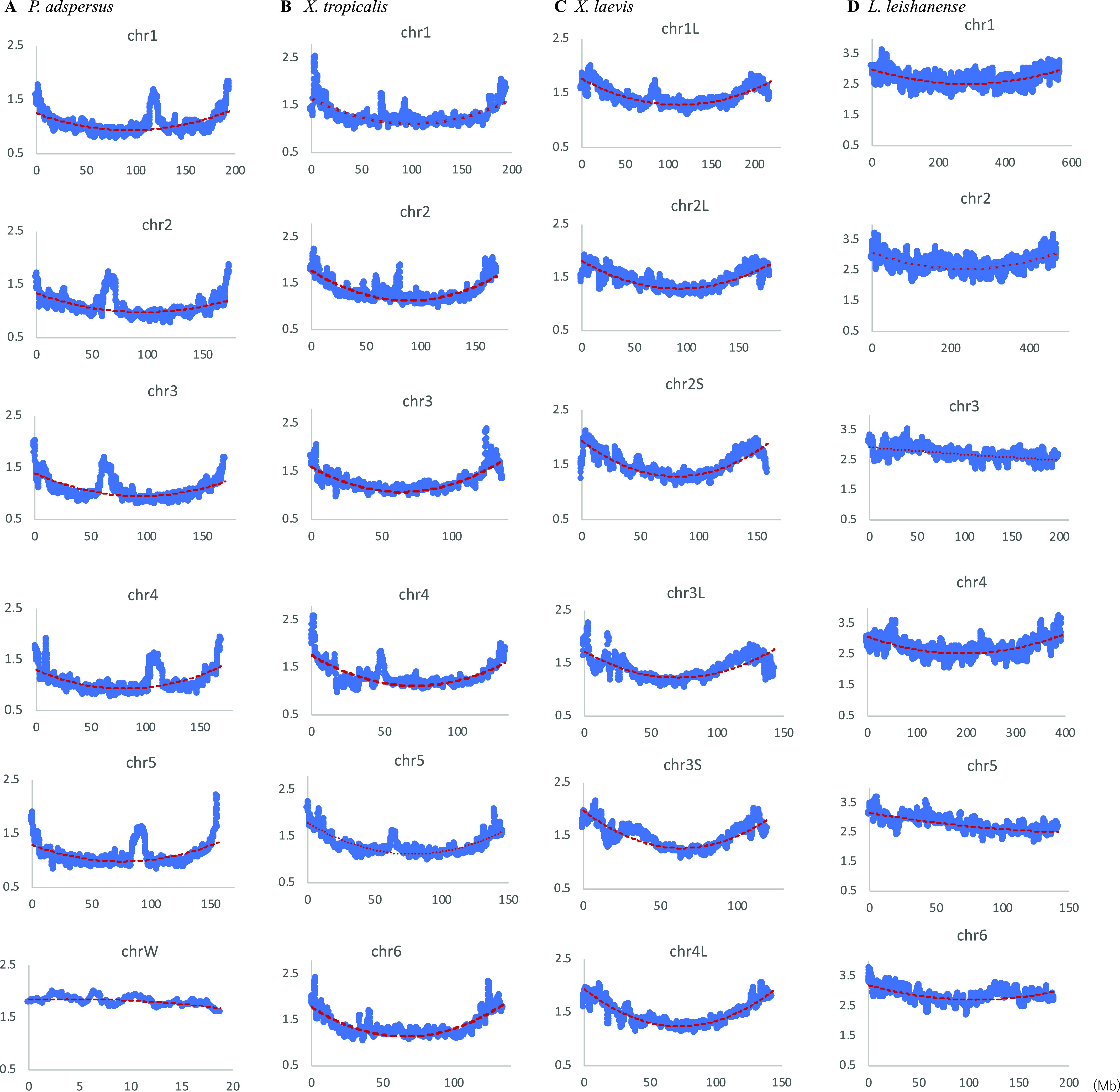
Distribution of CG composition in 1-Mb windows sliding with a 100-kb step on six chromosomes of four frogs. **(A)** *Pyxicephalus adspersu*s. **(B)** *Xenopus tropicalis*. **(C)** *Xenopus laevis*. **(D)** *Leptobrachium leishanense*. The binomial approximation is shown as a dashed line. In the graphs the x-axe shows positions in each chromosome (Mb), and the y-axe shows CG composition (%).

Fig 1Bii analyzed the dinucleotide composition in 1-Mb windows sliding with a 100-kb step, and using the same data set, we next plotted the CG composition (%) along chromosomes of four frogs whose genome sequences have been assembled into each chromosome. Fig 2A–D shows the pattern of the CG composition on six chromosomes for each frog including the W chromosome (chrW) in *P. adspersus*. The basal level on the *P. adspersus* chromosomes is approximately half of the level for the *L. leishanense* chromosomes. In most cases, a distinct increase in CG toward both ends of each chromosome is observed, and the highest peak is generally located near the end. This is particularly obvious for *P. adspersus* and *X. tropicalis*, which have the low CG frequency throughout the whole genome. In Fig 2, the binomial approximation is shown as a reddish brown dashed line. As mentioned above, chrW of *P. adspersus* is an evident exception (Fig 2A–D); whereas the chrW sequences used here do not include the pseudoautosomal region homologous to chrZ, there was no an increase at either end of chrW, but instead the whole region has the high CG.

Although lower than the prominent CG peaks at both ends of chromosomes, internal peaks are observed. This is particularly evident for *P. adspersus* (Fig 2A), and the biological significance of the internal peaks, which are often located close to the central area, will be discussed later in connection with centromeric and pericentromeric heterochromatins.

**Figure S4.**
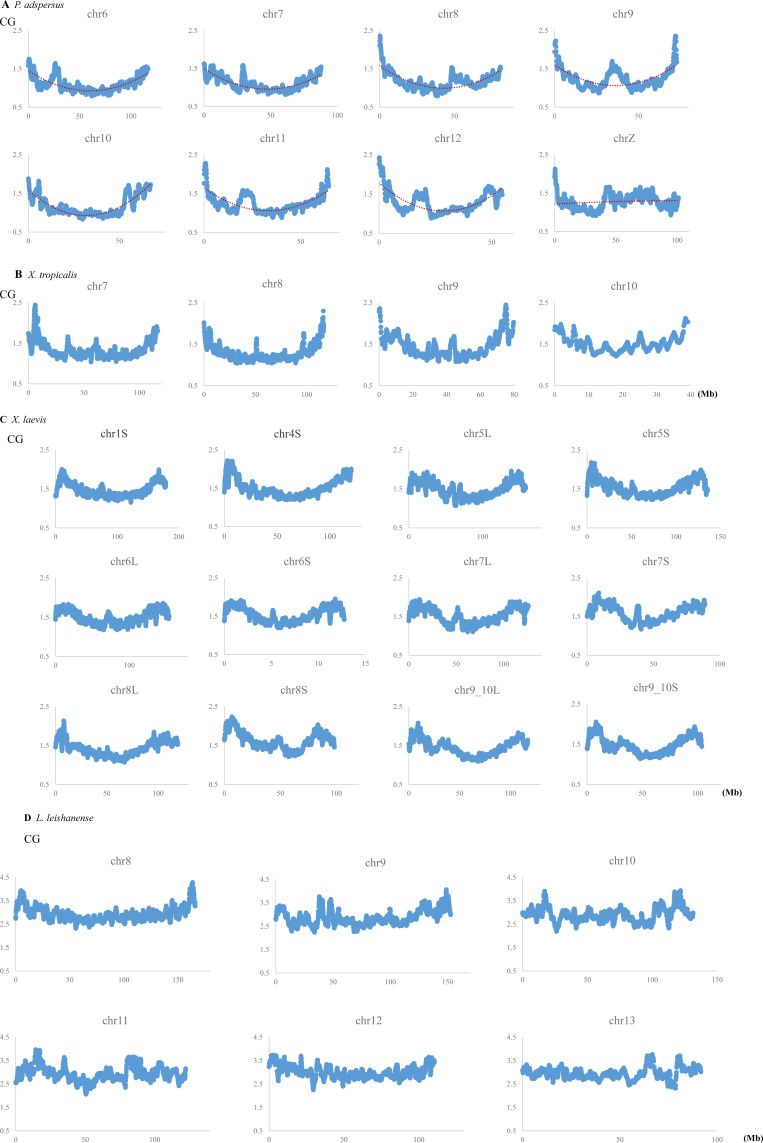
Distribution of CG compositions in 1-Mb windows sliding with a 100-kb step on chromosomes of four frogs. **(A)** *Pyxicephalus adspersu*s. To examine difference of the sex chromosome (chrZ) from autosomes, the binomial approximation is presented as a dashed line. **(B)** *Xenopus tropicalis*. **(C)** *Xenopus laevis*. **(D)** *Leptobrachium leishanense*.

CG distributions on all other chromosomes are presented in Fig S4A–D. In *P. adspersus*, downward parabolic patterns are observed for all autosomes, and chrZ has higher values in a relatively large internal area (Fig S4A). A distinct peak is observed at an internal position, often near the central area, of all chromosomes except chrW. In *X. tropicalis* and *X. laevis*, the same patterns are also observed for all 10 and 18 chromosomes, respectively, but a sharp decrease is seen very near the ends of all *X. laevis* chromosomes. For *L. leishanense*, with the lowest CG suppression at all chromosomes, downward parabolic patterns are observed for most chromosomes, but they tend to be weaker than for the other three frogs.

### Variation of CG suppression indexes along chromosomes

**Figure 3.**
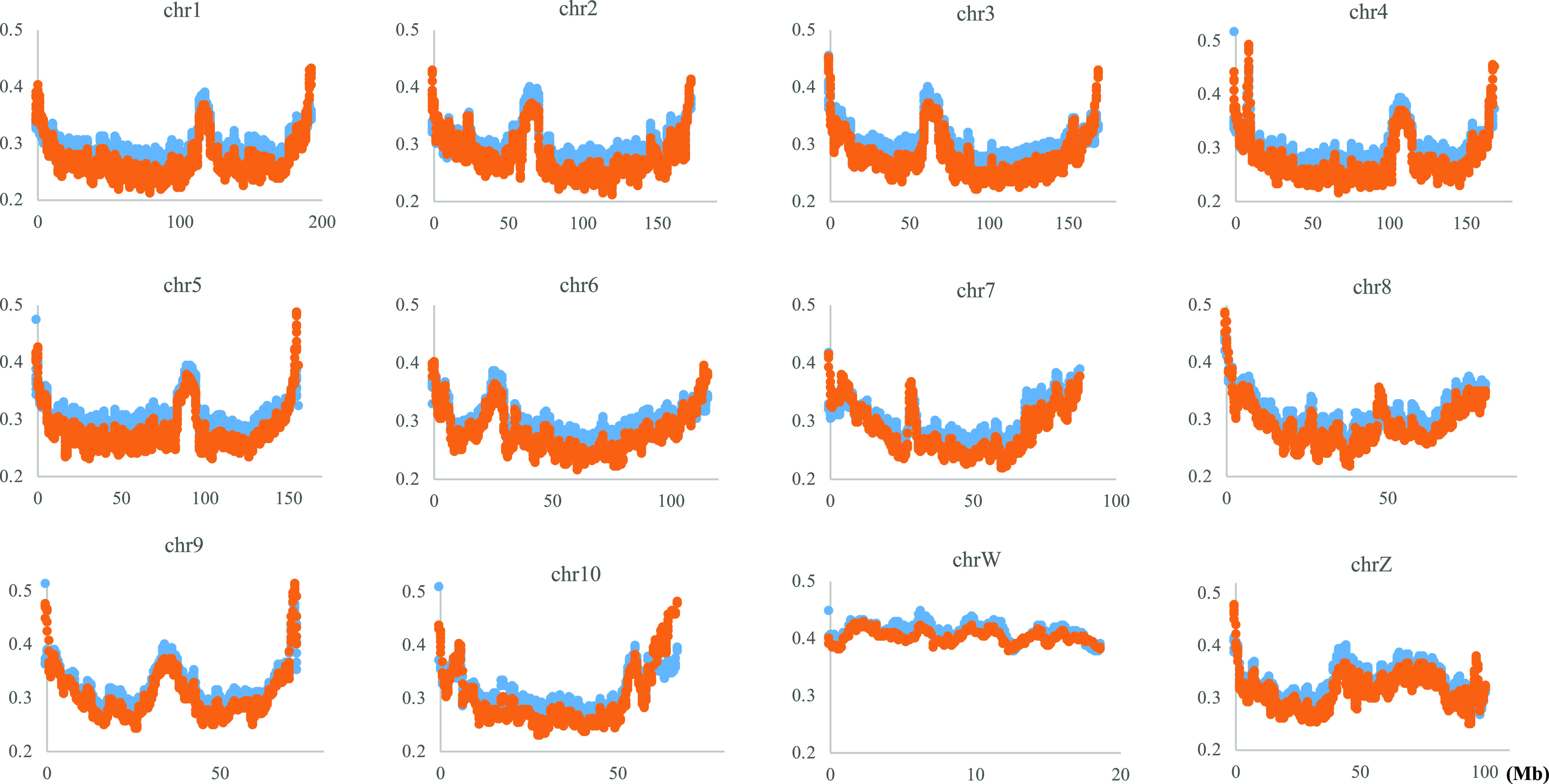
Distribution of the normalized CG (blue) and the CG/GC ratios (orange) on the *Pyxicephalus adspersus* chromosomes.

The variation of CG occurrences along the chromosome is inevitably influenced by variation in mononucleotide occurrences. Next, we examine whether the trend of increasing the frequency of CG toward both ends of chromosomes, as well as internal peaks that are prominent for *P. adspersus*, is a mere reflection of G+C% variation along chromosomes. Assuming that the CG variation is only the reflection of G+C% variation, the CG/GC ratio should fluctuate around 1.0 on the chromosome. Fig 3 plots two indexes of CG suppression, the CG/GC ratio (orange) and the Obs/Exp ratio of CG (blue), for *P. adspersus*, which has the most distinct parabolic distribution. Both indexes are clearly lower than 1.0 over the entire chromosome. The CG variation is therefore not reflected by the G+C% variation.

### Analysis of trinucleotide compositions

**Figure 4.**
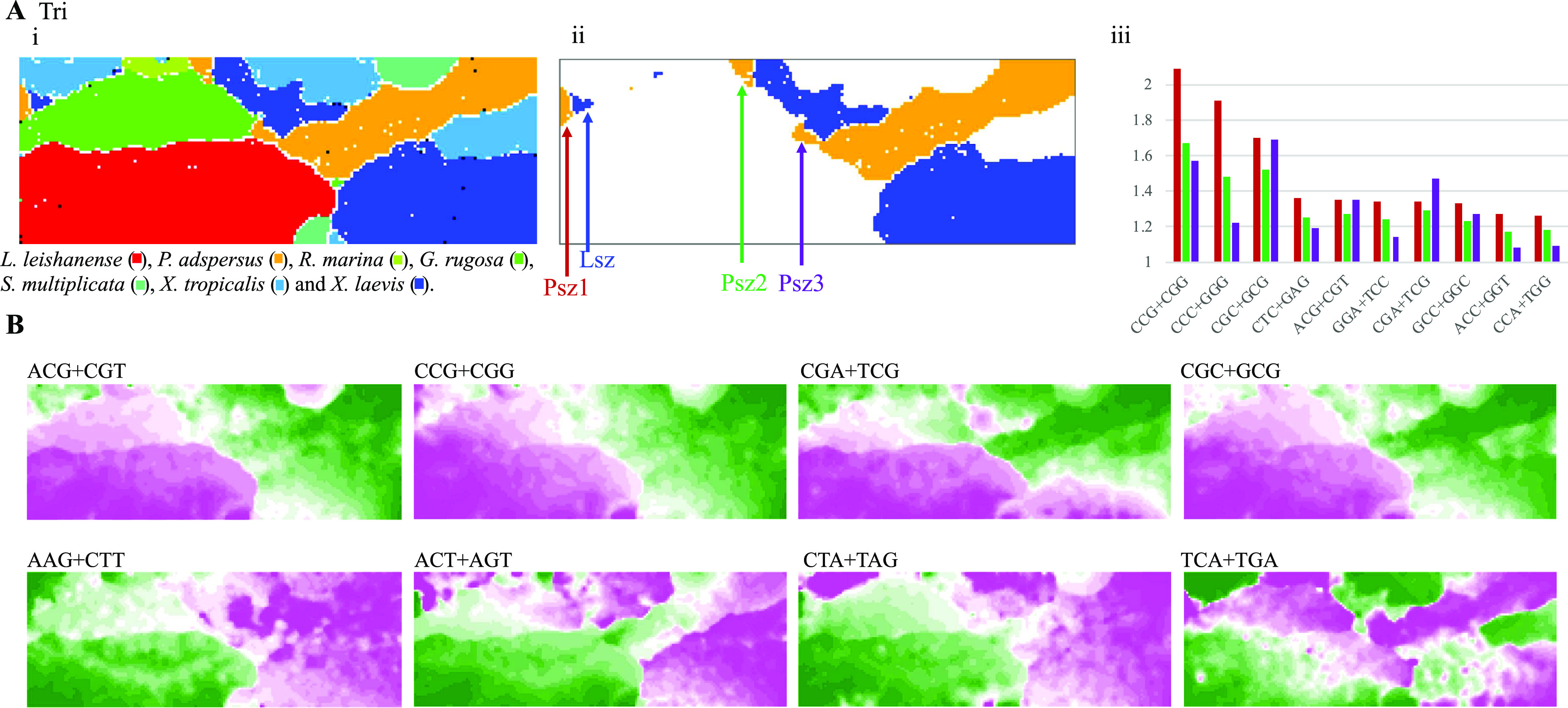
Trinucleotide (Tri) batch-learning self-organizing maps for 1-Mb sequences sliding with a 100-kb step. **(Ai)** Nodes are marked as described in Fig 1Ai. **(Aii)** Sequences of *Pyxicephalus adspersu*s and *Xenopus laevis* are displayed on the map, and their small satellite territories are marked by arrows. **(Aiii)** The ratio of occurrence of each of 10 trinucleotides in each satellite of *P. adspersus* to that in the entire genome is presented by a vertical colored bar: red (Psz1), green (Psz2), and violet (Psz3). Trinucleotides are arranged in the descending order of the ratio in Psz1, Psz2, and Psz3. For the Psz3 satellite, AGG+CCT was included in its top 10 instead of ACC+GGT, but the result of ACC+GGT is presented for comparison with other satellites. **(B)** The contribution level of each trinucleotide to each node on the map is visualized as described in Fig 1C; results of all trinucleotides are presented in Fig S6.

**Figure S5.**
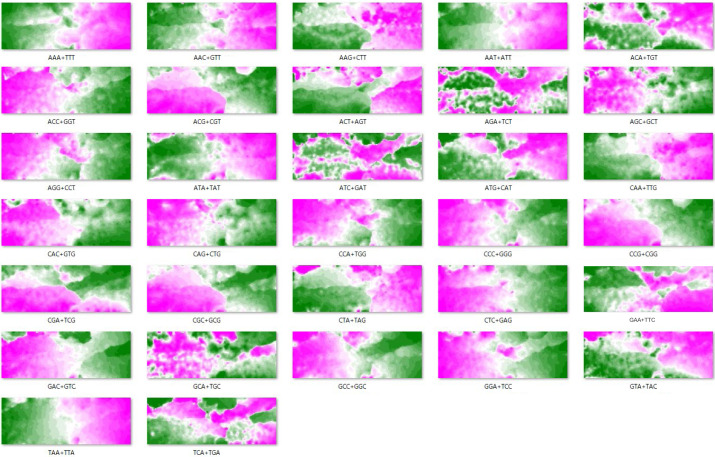
The contribution level of each trinucleotide to each node on the trinucleotide batch-learning self-organizing maps is visualized as described in Fig 3B.

Species-specific genome signatures become more pronounced as oligonucleotides become longer than dinucleotides (Abe et al, 2003, 2006). Fig 4Ai is a BLSOM for trinucleotide compositions, and its self-organization according to species becomes clearer than that for dinucleotide compositions. In Fig 4B, the degree of contribution of each trinucleotide to each node is displayed for eight examples; results of all trinucleotides are presented in Fig S5. The CG-containing trinucleotides (ACG+CGT, CCG+CGG, CGA+TCG, and CGC+GCG) occur more frequently (pink) in *L. leishanense* and *G. rugosa* than in other species, but, depending on the nucleotide added to CG, somewhat differential effects are observed for *G. rugosa* (Fig 4B, upper maps). The lower panels of Fig 4B show that three trinucleotides occur at low frequencies (green) in *L. leishanense*, but TCA+TGA appears at a higher frequency in *G. rugosa* than *L. leishanense*.

### Characterization of satellite territories of *P. adspersus* and *X. laevis*

On the dinucleotide BLSOM (Fig 1Bii), tiny territories of a particular species often scatter in large territories of other species, but they tend to be grouped into a small number of species-specific satellite territories on the trinucleotide BLSOM (Fig 4Ai). For *P. adspersus* and *X. laevis*, there are a few small satellites (e.g., those arrowed in Fig 4Aii) far away from their main territories, and three satellites of *P. adspersus* (Psz1, Psz2, and Psz3 arrowed in Fig 4Aii) are in contact with the large *G. rugosa* territory (green in Fig 4Ai). To characterize these satellites at the sequence level, we obtained sequences belonging to each satellite (Table S1) and then calculated their trinucleotide compositions and its ratio to that of its entire genome. The ratios of the top 10 trinucleotides for Psz1 are shown by red bars in Fig 4Aiii. The type of the top 10 trinucleotides for Psz2 (green) was the same as that for Psz1, but there was one difference for Psz3 (violet), as described in the legend of Fig 4Aiii. Considering their sequence types, not only the CG-containing trinucleotides but also the polypurine/polypyrimidine type (e.g., CCC+GGG, CTC+GAG, and GGA+TCC) also ranks high. The enrichment of the characteristic trinucleotides is likely a factor that creates satellite territories, and their differential abundance is probably a factor in creating different satellites.

Table S1 Start positions of 1-Mb sequences belonging to specific zones of *Pyxicephalus adspersus* (Pads) and *Xenopus laevis*.

**Figure 5.**
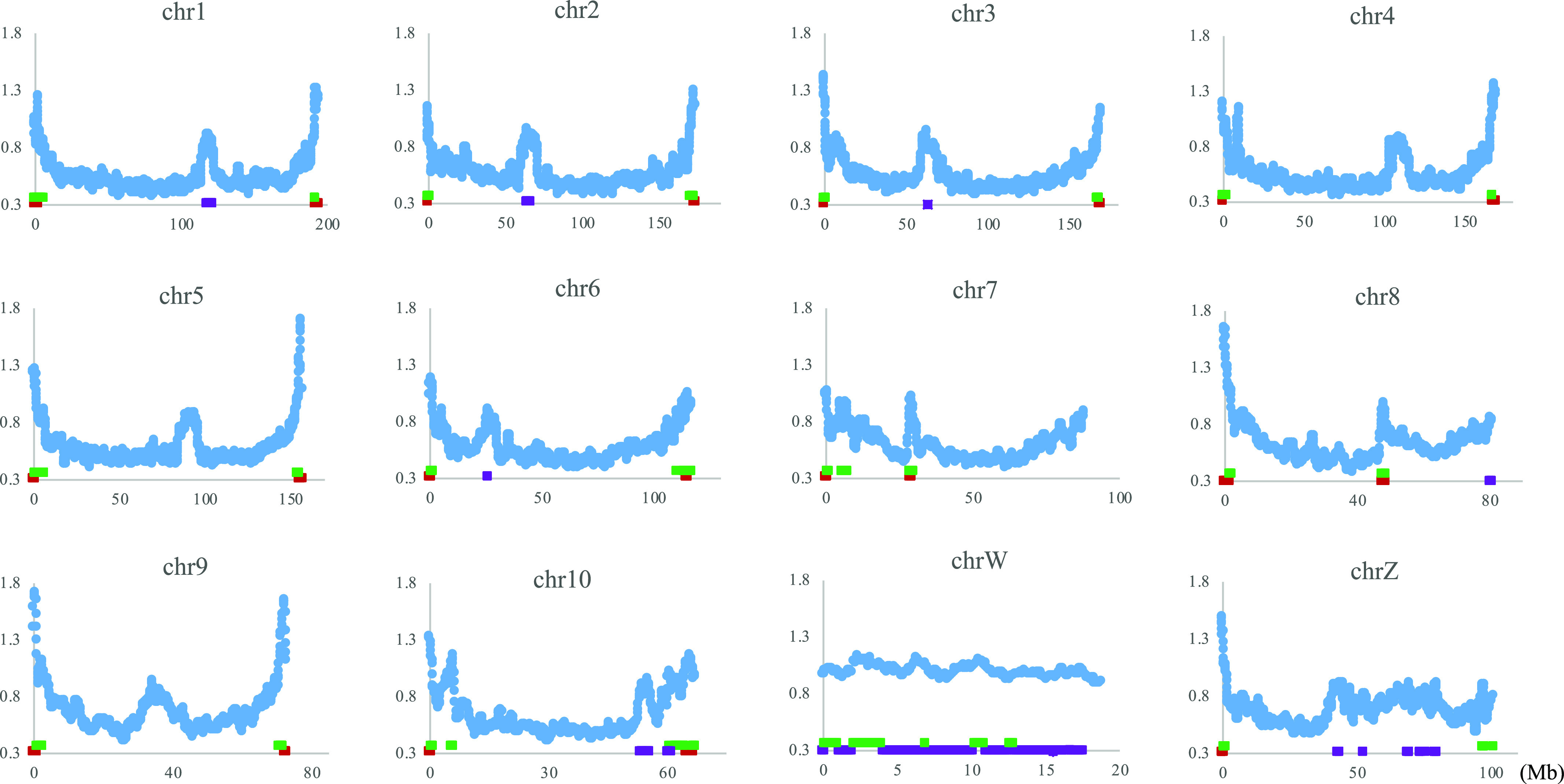
Distribution of CCG+CGG composition in 1-Mb windows sliding with a 100-kb step on the *Pyxicephalus adspersus* chromosomes. Chromosomal locations of sequences for Psz1, Psz2, and Psz3 are indicated by thick horizontal lines in red, green and violet on and above the x-axis. In the graphs, the x-axis shows positions in each chromosome (Mb), and the y-axis shows CCG+CGG composition (%).

For the *P. adspersus* chromosomes, Fig 5 analyzed the distribution of CCG+CGG, whose frequency is the highest in Psz1 and Psz2 and the second highest in Psz3. Similar to the CG distribution (Figs 1 and 2), the highest peak locates at or near the ends of all chromosomes except chrW. On and above the horizontal axis, chromosomal locations of sequences belonging to Psz1, Psz2 and Psz3 are indicated by red, green and violet marks, respectively. Psz1 sequences (red) are present on all chromosomes except chrW and mainly locate at the ends of most chromosomes. Psz2 sequences (green) also locate near the ends of most chromosomes but slightly inward from Psz1; on chrW, however, they widely scatter in internal areas. The Psz3 sequences (violet) are widely scattered across chrW and in multiple internal areas on chrZ, but locate in an internal peak on chr1, chr2, chr3, chr6, and chr10 and at one end of chr8.

When the same analysis was performed on the small *X. laevis* territory (Lsz in Fig 4Aii), the Lsz sequences were found at and near the ends of all chromosomes except chr9_10S (data not shown). When the ratio of the trinucleotide frequency of these sequences to that of the entire *X. laevis* sequence was calculated, the value for polypurine/polypyrimidine-type trinucleotides, CCC+GGG and CTC+GAG, was higher than for those containing CG, showing an obvious difference from *P. adspersus*.

### *G. rugosa* and sex chromosomal sequences

**Figure 6.**
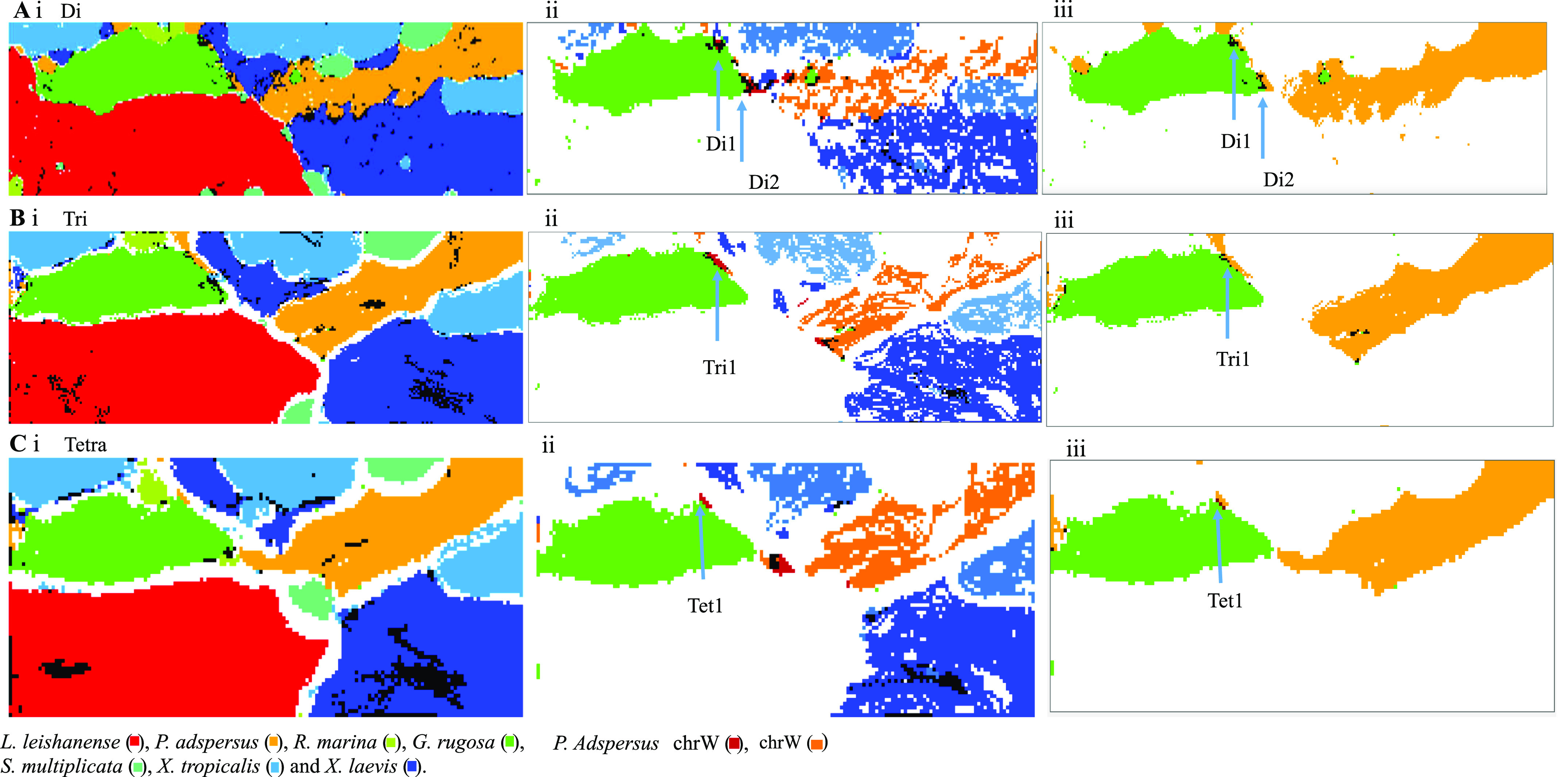
Batch-learning self-organizing maps (BLSOMs) for 1-Mb sequences sliding with a 10-kb step. **(Ai, Bi, Ci)** Di-, tri-, and tetranucleotide (Tetra) BLSOMs, respectively. **(Aii, Bii, Cii)** All sequences of *Glandirana rugosa* and sequences derived from sex chromosomes of other frogs are displayed: chrW (dark brown) and chrZ (light brown) of *Pyxicephalus adspersu*s, chr7 of *Xenopus tropicalis* (light blue), and chr2L of *Xenopus laevis* (dark blue). Satellite territories of *P. adspersus* (dark brown) around the *G. rugosa* territory are marked by arrows. **(Aiii, Biii, Ciii)** All sequences of *G. rugosa* and *P. adspersus* are displayed. The black nodes containing both *P. adspersus* and *G. rugosa* sequences are specified by Di1 and Di2 on the di- and trinucleotide BLSOMs (Aiii and Biii, respectively), and sequences belonging to these black nodes were extracted. The *G. rugosa* nodes in the vicinity of chrW are marked by dark brown (Tet1; Ciii) and sequences belonging to the nodes were extracted.

One reason for genome sequencing of *G. rugosa* is its intraspecies variability of sex chromosomal systems (Miura, 2007, 2017; Ogata et al, 2018). Using oligonucleotide BLSOMs, we next searched for *G. rugosa* sequences with a highly similar oligonucleotide composition to sex chromosomes of other frogs. On the trinucleotide BLSOM presented in Fig 4Ai, the species-dependent resolution was very high, and there were very few black nodes where *G. rugosa* sequences (green) overlap with those of other frogs, showing the resolution to be too high to identify sequences in search as black nodes. As a strategy for identifying the sequences with a highly similar oligonucleotide composition with sex chromosomes of other frogs, our previous method of sliding the 1-Mb window with 10-kb width (Wada et al, 2020) was considered to be suitable. In the BLSOM constructed for these sequence data, an average of 100 sequences per node was set (Fig 6). Even if one sequence of a certain species mixes with sequences of other species, the node is marked in black, and, therefore, overlapping of sequences with a highly similar composition among species will be detected as a black node. Furthermore, because of the very narrow sliding step, the composition in adjacent 1-Mb sequences becomes very similar, and sequences with a close genomic location should be visualized mainly as continuous dotted lines; that is, if there is a Mb-level structure with a highly similar composition between two species, this may be visualized as a black dotted line, rather than a single black dot. BLSOMs with di-, tri-, and tetranucleotide compositions are shown in Fig 6Ai, Bi, and Ci, respectively. Then, using these BLSOMs, all sequences of *G. rugosa* (green) and sequences derived only from sex chromosomes of other frogs are displayed (Fig 6Aii, Bii, and Cii); as the sex chromosome, chrW (dark brown) and chrZ (light brown) have been reported for *P. adspersus* (Denton et al, 2018 *Preprint*), and chr7 of *X. tropicalis* (light blue) and chr2L of *X. laevis* (dark blue) have been reported (Session et al, 2016; Mitros et al, 2019).

For chrW of *P. adspersus*, its sequence occupies only small zones (dark brown in Fig 6Aii, Bii, and Cii) which locate mainly around the *G. rugosa* territory. On the other hand, sequences of other sex chromosomes are widely distributed in the main territory of each species and are situated away from the *G. rugosa* territory. To precisely identify the overlap and proximity of *G. rugosa* and chrW in *P. adspersus*, all sequences of the two species are displayed (Fig 6Aiii, Biii, and Ciii). Di1 and D2 in Fig 6Aiii and Tri1 in Fig 6Biii show overlapped regions on di- and trinucleotide BLSOMs, respectively, and scaffold sequences of *G. rugosa* belonging to the black nodes were isolated. Although no overlap was seen on the tetranucleotide BLSOM, a series of chrW sequences were adjacent to the *G. rugosa* territory, as visualized as a dark-brown dotted line (Tet1 in Fig 6Cii), and the corresponding scaffold sequences were isolated.

### *G. rugosa* sequences with high sequence identity to chrW

**Figure 7.**
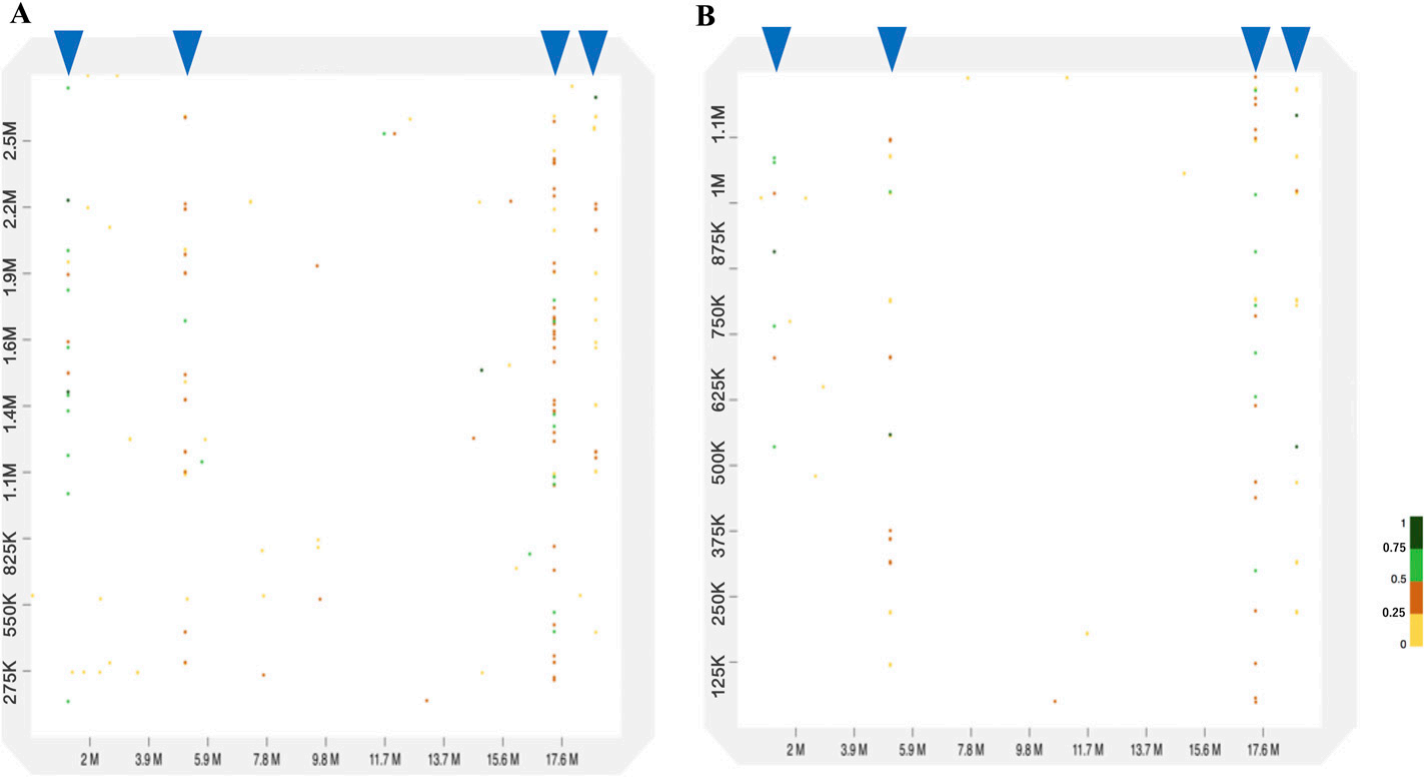
Dot plot analyses between chrW in *Pyxicephalus adspersus* and two scaffold sequences in *Glandirana rugosa*. **(A, B)** Each x-axis is the position of chrW, and the y axes are locations of (A) scaffold2393955 and (B) scaffold4079606. Blue arrowheads indicate the position of a series of dots in 1.5, 4.5, 17, and 18 Mb on chrW.

**Figure S6.**
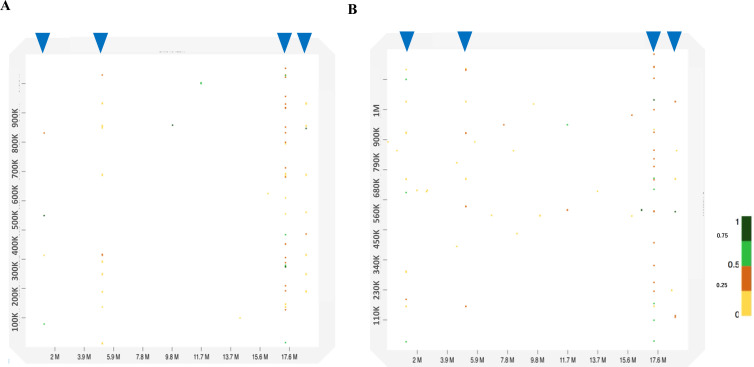
Dot plot analyses between chrW in *Pyxicephalus adspersus* and two scaffold sequences in *Glandirana rugosa*. **(A, B)** Each x-axis is the position of chrW, and the y axes are locations of (A) scaffold4871719 and (B) scaffold959090. Blue arrowheads indicate the position of dots in 1.5, 4.5, 17, and 18 Mb on chrW.

Of the isolated scaffold sequences from the three BLSOMs (Fig 6), four scaffold sequences of *G. rugosa* were commonly found in Di1, Tri1, and Tet1, and their sizes ranged from 1 to 2.7 Mb. To understand their overall similarity to chrW, a dot plot analysis (Cabanettes & Klopp, 2018) was performed; two patterns are presented in Fig 7A and B and the other two are presented in Fig S6A and B. For each scaffold, dots locate mainly around 1.5, 4.5, 17, and 18 Mb on chrW. For 17-Mb, dots with different levels of similarity are densely arranged across the entire area of each scaffold, but the actual density differs among scaffolds. As for other positions such as 18 Mb, dots appear less densely and primarily at different positions from the 17-Mb case for each scaffold. Overall, these scaffolds are filled with repetitive elements with different levels of identity to the chrW sequence that is located mainly at four different positions; the length of these elements was around 1–3.5 kb. To identify actual sequences of the repetitive elements, we next performed a BLASTn search (https://blast.ncbi.nlm.nih.gov/Blast.cgi?PAGE_TYPE=BlastSearch; Altschul et al, 1990) of the four scaffold sequences against the chrW sequence. The highest identity was 85% for 3.4 kb, and many sequences with different levels of identity were found for each scaffold; examples of sequence alignments are presented in Supplemental Data 1–4.

Supplemental Data 1. The example of sequence alignments in the BLASTn search. The BLASTn search was performed using the *G. rugosa* scaffolds against the chrW sequences of *P. adspersus.* The example includes scaffold 2393955 sequences which shows the highest identity against the chrW.

Supplemental Data 2. The example of sequence alignments in the BLASTn search. The BLASTn search was performed using the *G. rugosa* scaffolds against the chrW sequences of *P. adspersus.* The example includes scaffold 2393955 sequences which shows the highest identity against the chrW.

Supplemental Data 3. The example of sequence alignments in the BLASTn search. The BLASTn search was performed using the *G. rugosa* scaffolds against the chrW sequences of *P. adspersus.* The example includes scaffold 2393955 sequences which shows the highest identity against the chrW.

Supplemental Data 4. The example of sequence alignments in the BLASTn search. The BLASTn search was performed using the *G. rugosa* scaffolds against the chrW sequences of *P. adspersus.* The example includes scaffold 2393955 sequences which shows the highest identity against the chrW.

**Figure S7.**
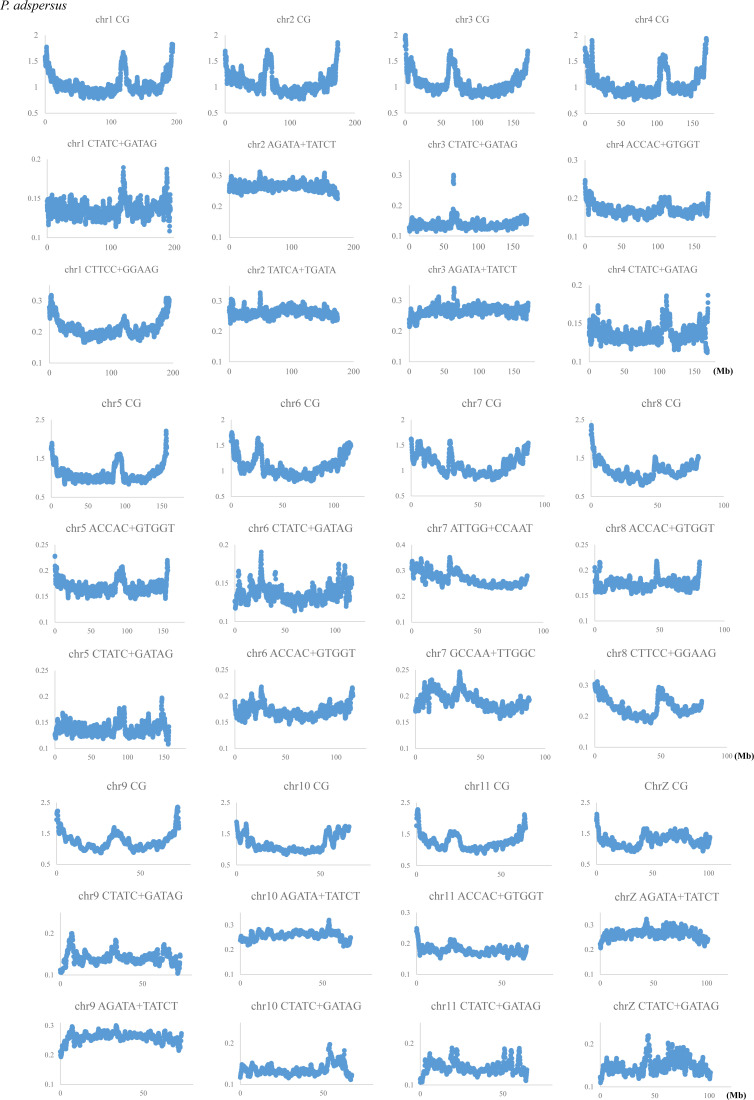
Distribution of core elements of transcription factor binding sequences in 1-Mb windows sliding with a 100-kb step on chromosomes of *Pyxicephalus adspersus*. For reference, CG distribution is presented.

A portion of the homologous sequences are 86–92% identical to a Bam transposon (520 bp) that was found only in the Ranidae family by the BLASTn search (Casola et al, 2004; Supplemental Data S5), and the Bam transposon is C/G-rich and similar to the large transposon element hAT (*hobo-Activator*-*Tam3*) superfamily (Ragghianti et al, 2004). The hAT occupied 5.9% of the *G. rugosa* genome (Fig S7), and the Bam sequence has a high CG frequency (33%). *G. rugosa* and *P. adspersus* are Ranidae family members, and a total of 250 scaffolds in the *G. rugosa* genome have 1,018 Bam copies (>82% identity). *P. adspersus* has 75 Bam copies (>81% identity), and chrW includes four copies and are highly similar to the four scaffolds in *G. rugosa* as shown in the dot plots (Figs 7A and B and S6A and B). The Bam sequences are thought to have increased in *G. rugosa* after its divergence from *P. adspersus* 89 million years ago (MYA) (http://www.timetree.org), and the burst of transposons may have caused the increase in both size and CG composition in the whole *G. rugosa* genome.

Supplemental Data 5. The sequence alignment of the chrW (*P. adspersus*) and transposon dalBam (*Rana dalmatina*) in the BLASTn search.

Putting these observations together, *G. rugosa* has Mb-level structures rich in kb-level repetitive elements that are homologous to the chrW sequences (e.g., the Bam transposon), and this should provide fundamental knowledge for future studies of its sex chromosomes. On the BLSOMs displayed in Fig 5, there are additional regions, where sex chromosomal sequences of different species are overlapping, and we are undertaking a separate detailed study for characterizing these sequences.

## Discussion

As the strategy for this data-driven study, knowledge discovery was left to the unsupervised and explainable AI, BLSOM. Then, based on the obtained knowledge, successive detailed analyses were conducted. We next discuss the biological significance of the obtained findings.

### CG suppression

The level of CG suppression is highly conserved among mammals but varies substantially among frogs (Figs 1D and S3); for example, even for *G. rugosa* and *P. adspersus*, the values are 0.45 and 0.30, respectively. *G. rugosa* and *P. adspersus* are diploid species and evolutionarily close to each other, but their assembled genome sizes differ greatly: *G. rugosa*, 7.08 Gb (this study), and *P. adspersus* (1.56 Gb: Denton et al, 2018 *Preprint*). The difference in the CG suppression may relate to the difference in genome sizes because numerous transcripts potentially present in large genomes should be severely suppressed in a very wide range of the genome, and their chromatin should therefore be highly condensed and heterochromatinized Grewal & Jia, 2007). When C in CG undergoes methylation, the methylated CG tends to mutate to TG/CA, resulting in the CG suppression (i.e., CG deficiency) (Klose et al, 2005). However, if proteins bind stably to the DNA, the mutation is suppressed, and therefore, in constitutive heterochromatin regions, CGs are well conserved, resulting in the weaker CG suppression (Klose et al, 2005; Bogdanović & Veenstra, 2009). This explanation is only one hypothesis, and other mechanisms such as involvement of ncRNA may work in suppressing gene expression in a wide genomic area.

### Generality and exception

When most chromosomes of a certain species have a certain common characteristic, focusing on the exceptional chromosome should provide valuable information about the chromosome. The CG frequency does not have high peaks near the ends of chrW but is maintained at a high level over the entire area (Fig 2A), and this exceptional feature resembles human chrY (Wada et al, 2015, 2020) of which a large portion is composed of constitutive heterochromatin. This suggests that a large portion of chrW, as well as the four *G. rugosa* scaffold sequences mentioned above, is composed of constitutive heterochromatin.

In the CG distribution on frog chromosomes (Figs 2 and S4), downward parabolic patterns are observed for almost all chromosomes, with the evident exception of chrW. Taking together the continuously changing parabolic pattern across each chromosome and the generality for all frogs, this characteristic is thought to relate to global features of chromosomal DNA segments, such as their nuclear locations: nuclear envelope side, internal side and so on. Signal sequences responsible for the nuclear organization may not be embedded in a few narrow chromosomal regions but distributed in broader ones (Bernardi, 2019).

In addition to the distribution of CG in Figs 2 and S4, Fig 5 shows the CCG+CGG distribution for almost all *P. adspersus* chromosomes, and therefore, exceptional cases can be easily explained. On all autosomes, the continuously changing parabolic pattern is seen, but in chr7 and chr8, somewhat discontinuous changes appear at an internal peak site (Figs 5 and S4). Furthermore, Psz1 and Psz2 sequences (red and green bars, respectively), which locate primarily at the ends of other autosomes, locate at the internal sites of chr7 and chr8; and Psz3 sequences (violet), which locate at the internal peak of other autosomes, locate at one end of chr8 (Fig 5). These irregularities may reflect an intra- or interchromosomal rearrangement, such as via the telomere and centromere.

### Similarity of Mb-level peaks of frogs and human

This study mainly analyzed CG dinucleotide and CG-containing trinucleotides, and found Mb-level structures rich in these oligonucleotides. We next discuss the internal peaks most evidently observed on the *P. adspersus* chromosomes. Our previous Mb-level analyses on the human chromosomes found high CG peaks (a Mb-level CpG island) in centromeric and pericentromeric constitutive heterochromatin regions of all chromosomes except chrY (Wada et al, 2015, 2020). Those studies also analyzed oligonucleotides longer than pentanucleotides and found that a wide range of transcription factor binding sequences (TFBSs) were enriched in Mb-level CpG islands located in the centromeric and pericentromeric regions (Iwasaki et al, 2013; Wada et al, 2020); specifically, we analyzed a total of ∼5,000 TFBSs of hexa-to octanucleotides compiled by the SwissRegulon Portal (Pachkov et al, 2013) and found that the enriched TFBS types differ depending on the chromosome.

The present frog genome study focused on mono- to tetranucleotides, and TFBS occurrences cannot be directly discussed. For longer oligonucleotides, we have been conducting a separate detailed study but have preliminarily analyzed eight pentanucleotides that are consensus core elements for a wide variety of vertebrate TFBSs (Iwasaki et al, 2013; Fornes et al, 2020) and found that the TFBS core elements, such as GATA-containing or polypurine/polypyrimidine-type pentanucleotides, are evidently enriched in the internal Mb-level CpG islands of *P. adspersus* (Fig S7). When considering similarity to the human chromosomes, the internal Mb-level CpG islands of *P. adspersus* are surmised to correspond to the centromeric and pericentromeric heterochromatin regions enriched in TFBSs.

**Figure S8.**
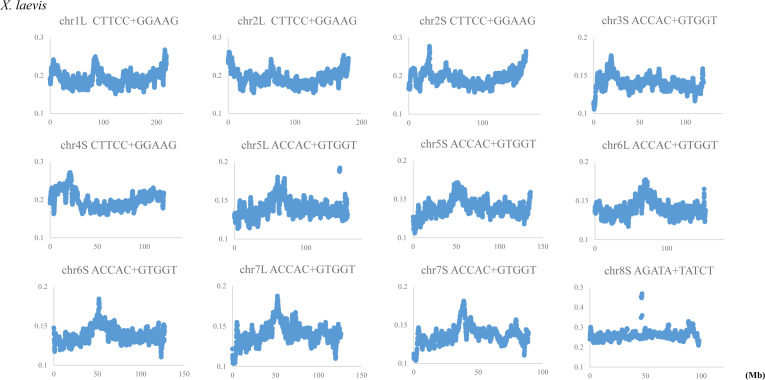
Distribution of core elements of transcription factor binding sequences in 1-Mb windows sliding with a 100-kb step on chromosomes of *Xenopus laevis*.

If this idea is correct, it would seem contradictory that there are few internal Mb-level CpG islands on the *X. laevis* chromosomes (Fig 2). An additional analysis on its chromosomes for the above TFBS cores showed a distinct internal peak which mainly locates close to the central area of each chromosome and is enriched for the TFBS cores such as a polypurine/polypyrimidine-type pentanucleotide, CTTCC+GGAAG (Fig S8). In the case of *X. laevis*, CG occurrences are ubiquitously higher than for *P. adspersus*, and, therefore, various TFBSs, rather than CG-containing oligonucleotides, may be specifically enriched in its centromeric and pericentromeric heterochromatin regions as clearly marking the functionally important structure.

### Perspectives on the evolution of sex chromosomes

Sex chromosomes are not well developed in the frogs analyzed here, except in *P. adspersus* which has 12 autosomes and a pair of heteromorphic sex (ZW) chromosomes (Denton et al, 2018 *Preprint*). *G. rugosa* has 13 chromosomes, and chr7 is a homomorphic sex chromosome (Miura, 2017). The sex chromosomes in these two frogs are believed to have diverged at least ∼89 MYA, and they have three common sex chromosomal genes: SRY-box 3 (*SOX3*), α thalassemia/mental retardation syndrome X-linked (*ATRX*), and androgen receptor (Miura, 2017; Denton et al, 2018 *Preprint*). These genes are candidates for a sex-determining gene in *G. rugosa* and *P. adspersus* and are also located on the human sex chromosomes. *SOX3* is an X-linked homologous gene of a sex-determining region Y (*SRY*) gene which is a therian male determiner and diverged from *SOX3* ∼160 MYA (Katsura et al, 2018). In addition, *ATRX* is located on the sex chromosome in the Mexican axolotl (*Ambystoma mexicanum*), and the W-linked gene (*ATRW*) is a candidate of the sex-determining gene (Keinath et al, 2018). These observations suggest the convergent molecular evolution that some similar characteristics have been obtained on the sex chromosomes in therians and amphibians independently.

Sex-determining systems differ among frogs. In *X. laevis*, a female determiner, the W-linked double-sex and mab-3 (DM) domain gene (*Dm-W*), has been identified (Yoshimoto et al, 2008). *Dm-W* was duplicated from the DM related transcription factor 1 gene (*DMRT-1*), and *DMRT-1* is known to be a master sex-determining gene in several vertebrates (Bachtrog et al, 2014; Miura, 2017). In *G. rugosa* and *P. adspersus*, however, *DMRT-1* is on an autosome and is probably not the sex-determining gene. The Mb-level structures of *G. rugosa* that are rich in kb-level repetitive elements homologous to chrW sequences of *P. adspersus* should provide fundamental knowledge for future studies of sex chromosomes of these species.

### Perspectives on BLSOM analyses of comparative genomics

**Figure S9.**
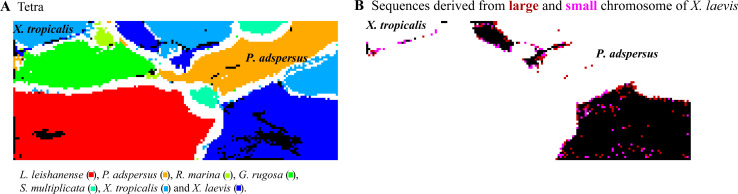
Tetranucleotide (Tetra) batch-learning self-organizing map (BLSOM) for 1-Mb sequences sliding with a 10-kb step. **(A)** This BLSOM is the same to that presented in Fig 5Ci. **(B)** If a node of the BLSOM contains sequences derived from large or small chromosomes of *Xenopus laevis*, it is shown in dark brown or pink, respectively. If a lattice point contains both chromosomes, it is shown in black. The *X. laevis* sequences located at the margins of a *Xenopus tropicalis* territory were mainly derived from the small chromosome, whereas those within the *Pyxicephalus adspersus* territory were all derived from the large chromosome.

In the present study, we focused on mono-to tetranucleotide compositions primarily in 1-Mb fragments and conducted comparative genome analyses using different species. Here, we discuss its usefulness for characterizing an intraspecies difference. As a preliminary analysis, we examined whether the large and small chromosomes of *X. laevis*, which were derived from a heteroploid of two different species (Session et al, 2016), were separated on the tetranucleotide-BLSOM presented in Fig 6C. Whereas the separation between large and small chromosomes of *X. laevis* was poor in its major territories, the sequences that invaded other species’ territories showed clear differences between the large and small chromosomes; for example, sequences that invaded the *P. adspersus* territory were derived primarily from the large chromosome of *X. laevis* (Fig S9). This indicates usefulness of the BLSOM analysis for identifying intraspecies differences.

The present study showed that the genome signature was different among frogs and that the oligonucleotide compositions on chrY and chrW were similar beyond species. In future, details of the genome and sex chromosomal evolution will be better described by various BLSOM tools after the completion of detailed sequencing of the *G. rugosa* genome.

## Materials and Methods

### Genome sequencing and de novo assembly

We extracted DNA from a *G. rugosa* male (ZZ) individual collected in Kyoto (Ogata et al, 2018). One paired-end library (insert size, 300) and four mate-pair libraries (insert size, 3,000, 6,000, 10,000, and 15,000) were prepared using a TruSeq DNA PCR-Free LT Sample Prep Kit and a Nextera Mate Pair Sample Prep Kit, respectively. Sequencing was performed using Illumina HiSeq 2500 and NovaSeq 6000 (Table S2). Adapters and low-quality regions in reads were trimmed using Platanus_trim 1.0.7 (http://platanus.bio.titech.ac.jp/pltanus_trim) with the default parameters. The assembled genome size and heterozygosity were estimated by GenomeScope software (Vurture et al, 2017) after inputting the trimmed paired-end reads, and the estimated coverage depth was 154 (paired-ends, 117; mate-pairs, 37). De novo assembly was performed using Platanus-allee 2.2.2 (Kajitani et al, 2019) with the default parameters except for those related to multi-threading (-t 80) and maximum memory-usage (-m 2048). Note that only the paired-end library was input into the contig-assembly (“platanus_allee assmble” command), whereas all libraries were used for the following steps (“platanus_allee phase” and “platanus_allee consensus” commands). The sequence of the mitochondrial genome was separately constructed by a manual curation. Finally, short sequences (<500 bp) were discarded from the consensus (haploid) scaffolds set, and fragmented mitochondrial sequences were replaced using the BLASTn search (Altschul et al, 1990).

Table S2 Statistics of whole genome sequencing reads of *Glandirana rugosa*.

**Figure S10.**
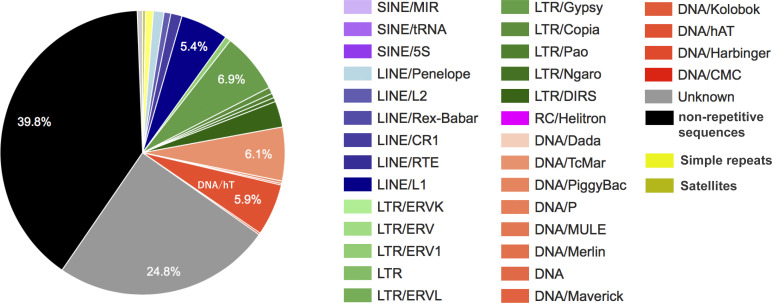
The occurrence ratios of repetitive sequences in the *Glandirana rugosa* genome.

We detected the repetitive sequences using RepeatModeler2 (Flynn et al, 2020) and RepeatMasker (Smit et al, 2013–2015; http://www.repeatmasker.org), and the occurrence ratios of each repetitive sequence to the whole genome are shown in Fig S10.

### BLSOM

A self-organizing map (SOM) is an unsupervised algorithm that projects high-dimensional data nonlinearly onto a two-dimensional plane (Kohonen et al, 1996), and Kanaya et al (2001) modified Kohonen’s SOM for genome informatics to make the learning process and resulting map independent on the order of data input on the basis of a batch-learning SOM. In Kohonen’s original SOM, the initial vectorial data were set by random values, but in BLSOM, the initial vectors were set based on the widest scale of the sequence distribution in the oligonucleotide frequency space with principal component analysis. Weights in the first dimension (*I*) were arranged into lattices corresponding to a width of five times the SD (5σ1) of the first principal component: the second dimension (*J*) was defined by the nearest integer greater than σ2/σ1 × *I*; and *I* was the average number of sequence data per node. σ1 and σ2 were the standard deviations of the first and second principal components, respectively. The weight vector on the *ij*^th^ lattice (*w*_*ij*_) was represented as follows (*i* and *j* represent the position of lattice points):

$$W_{i,j}=x_{\mathrm{av}}+\frac{5\sigma_{1}}{I}\left[b_{1}\left(i-\frac{I}{2}\right)+b_{2}\left(j-\frac{J}{2}\right)\right],$$

where *x*_*av*_ is the average vector for oligonucleotide frequencies of all input vectors, and *b*_*1*_ and *b*_*2*_ are eigenvectors for the first and second principal components. Weight vectors (*w*_*ij*_) were set and updated as described previously (Abe et al, 2003).

Because principal component analysis can grasp basic properties of genomic sequences such as G+C%, the global patterns of oligonucleotide BLSOMs, in which various learning parameters and the number of sequences per node are changed, resemble each other (Abe et al, 2003, 2005). The BLSOM for oligonucleotide composition was constructed as described by Abe et al (2003), and oligonucleotides diagnostic for category-dependent separation were visualized as described by Kanaya et al (2001). The BLSOM program can be obtained from a GitHub repository (https://github.com/yukakokatsura/BLSOM).

## Data Availability

The DNA read libraries and the genome assembly of *G. rugosa* have been deposited at the DNA Data Bank of Japan (DDBJ) Sequence Read Archive (DRA009996) under BioProject PRJDB9666. Genome sequences of six frogs were obtained from the following ftp sites and accession numbers: *L. leishanense* (https://ftp.ncbi.nlm.nih.gov/genomes/all/GCA/009/667/805/GCA_009667805.1_ASM966780v1/), *P. adspersus* (CM016416-CM016429), *X. tropicalis* (http://ftp.xenbase.org/pub/Genomics/JGI/Xentr10.0/), *X. laevis* (http://ftp.xenbase.org/pub/Genomics/JGI/Xenla9.2/), *R. marina* (ftp://parrot.genomics.cn/gigadb/pub/10.5524/100001_101000/100483/canetoad.v2.2.fasta.gz) and *S. multiplicata* (VKOC01000001-VKOC01049736).

## Supplementary Material

Reviewer comments
